# Endobronchial embolization for life-threatening hemoptysis with Endobronchial Watanabe Spigot

**DOI:** 10.1186/s13104-017-2635-4

**Published:** 2017-07-21

**Authors:** Sze Shyang Kho, Swee Kim Chan, Mei Ching Yong, Siew Teck Tie

**Affiliations:** Department of Medicine, Respiratory Medicine Unit (RCU), Sarawak General Hospital, Jalan Hospital, 93586 Kuching, Sarawak Malaysia

**Keywords:** Massive hemoptysis, Endobronchial embolization, Endobronchial Watanabe Spigot

## Abstract

**Background:**

Massive hemoptysis is a common encounter in respiratory medicine. Bronchoscopy plays an important role in localizing the origin of bleeding, as well as endoscopic treatment of centrally located lesions. Endobronchial embolization is a novel technique enabling the management of hemoptysis arising even from peripheral lesions, via occlusion of the culprit bronchus, thereby securing the airway. Endobronchial Watanabe Spigot had been advocate in the treatment of bronchopleural fistula and the use of this novel technique had since then been expanded into the management of massive hemoptysis. To the best of our knowledge, this is the first reported case in Malaysia.

**Case presentation:**

78-year-old lady who presented with life-threatening hemoptysis leading rapidly to cardiac arrest upon arrival. Spontaneous circulation was restored after resuscitation with an urgent thoracic computed tomography angiogram revealed bleeding likely from the posterior basal segment of left lower lobe, with bronchiectatic changes. Urgent flexible bronchoscopy revealed airway flooding, with bleeding originating from the lingular and posterior-basal segment of the left lower lobe. Airway toileting was performed and two 7 mm Endobronchial Watanabe Spigots were plugged into the culprit bronchi. Urgent bronchial artery embolization was then attempted, but was unsuccessful. She was managed conservatively, as surgical resection was deemed high risk. The spigots were removed 4 days later uneventfully. There was no recurrence of hemoptysis, and patient remained well during 1-month follow up.

**Conclusions:**

The utmost priority in managing life-threatening hemoptysis is to prevent airway flooding. Endobronchial embolization with Endobronchial Watanabe Spigot is useful as a temporary measure before definitive therapy, or can itself be the main therapeutic player in the hemoptysis armament for high-risk patients.

## Background

Massive hemoptysis is one of the most challenging conditions in respiratory medicine which requires prompt intervention to prevent morbidity and mortality. Flexible bronchoscopy plays a pertinent role in localizing the origin of bleeding and to administer endoscopic treatment if the culprit lesion is centrally located [1]. However, bronchoscopy had curative limits if bleeding arose from peripheral lesions. Endobronchial embolization using Endobronchial Watanabe Spigot (EWS) had initially shown promise in the management of bronchopleural fistulas [2], and the use of this novel technique had since then been expanded into the management of massive hemoptysis, including the previously difficult peripheral lesions, with a success rate of 78% [3]. We report a case of life-threatening hemoptysis managed conservatively by endobronchial embolization with EWS. To the best of our knowledge, this is the first reported case in Malaysia.

## Case presentation

A 78-year-old lady with no prior known chronic medical illness, presented with sudden onset of hemoptysis on the day of admission. She denied fever, cough or any chest pain prior. She was tachypneic upon arrival with 90% oxygen saturation on room air. Preliminary investigation revealed haemoglobin level of 13.5 g/dl, normal coagulation profile, and type 1 respiratory failure on arterial blood gas, with bibasal alveolar infiltrates on chest X-ray (CXR). She developed another bout of hemoptysis in the emergency unit and went into cardiac arrest. She was resuscitated and intubated; and fresh blood was noted upon suction from the size 7 mm endotracheal tube.

**Fig. 1:**
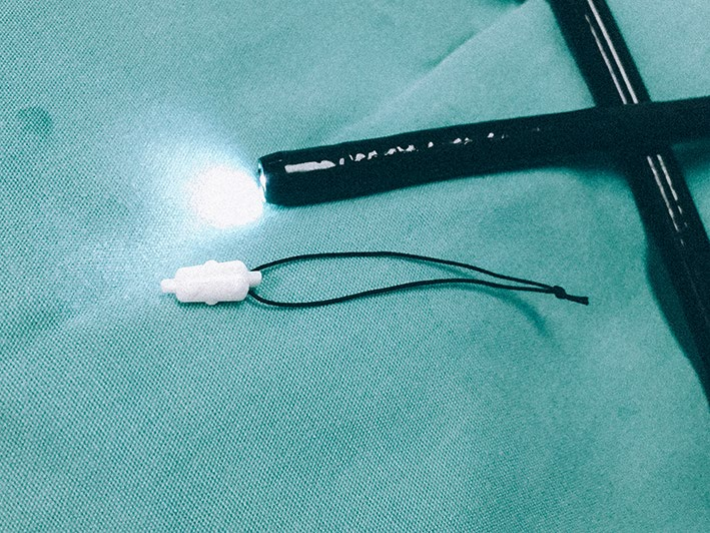
A nylon thread was sutured to the distal end of the EWS for ease of forceps retrieval in the event of inadvertent grip-loss during insertion

**Fig. 2:**
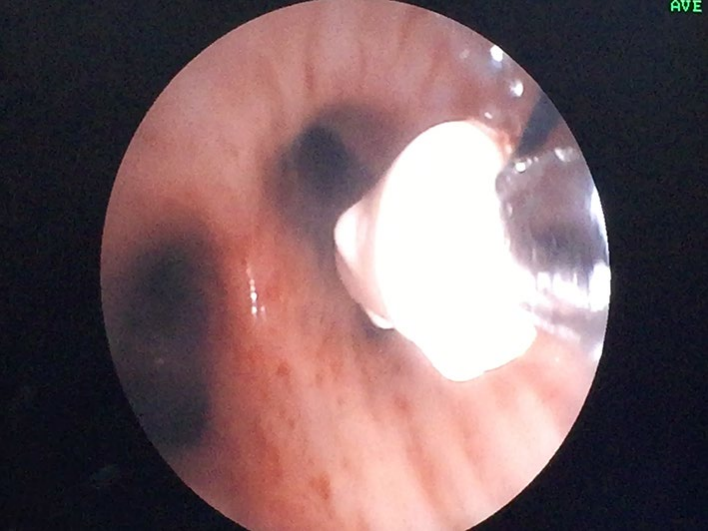
The bronchoscope with the grasped EWS was inserted as a single unit, and inserted firmly into the target bronchi under direct vision

**Fig. 3:**
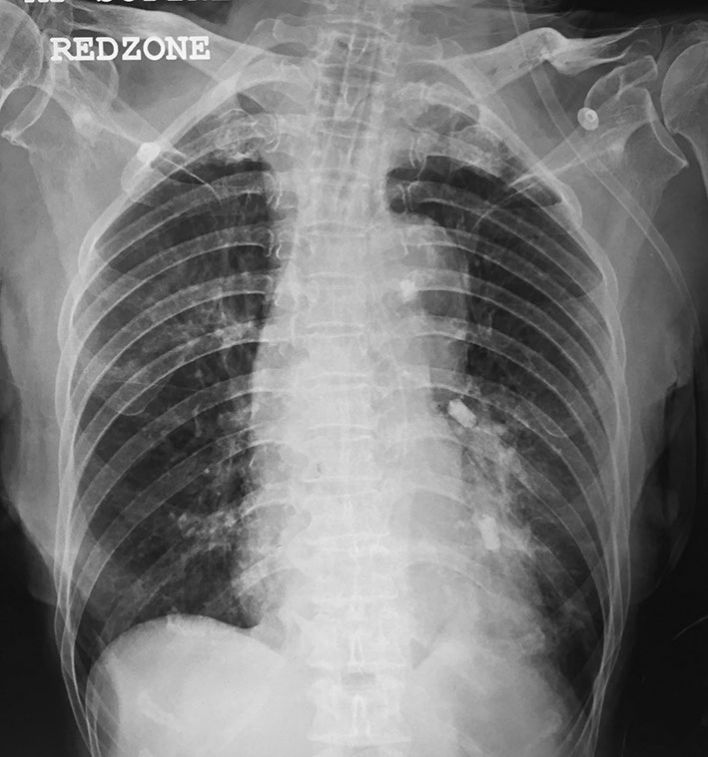
Post procedure CXR showed two EWS in optimal positions

Urgent thoracic computed tomography (CT) angiogram revealed a focal wedge-shaped consolidation at the posterior-basal segment of the left lower lobe and lingular lobe with surrounding bronchiectatic changes; with dilatation of the inferior pulmonary vein—suggesting hyperemia—but with no active contrast extravasation. Endotracheal aspirate sent urgently for Acid-fast bacilli and Xpert MTB/RIF was negative. She was started empirically on broad-spectrum antibiotic and her endotracheal tube was changed to 8 mm by the anesthesiologist to allow greater leeway for bronchoscopic intervention. Emergency bronchoscopy was performed using a flexible therapeutic bronchoscope (*Fujinon EB530*-*XT*), and revealed airway flooding with blood clots. All blood clots were evacuated, with no central intraluminal lesion noted. However, the bleeding source was localized to lingula and posterior-basal segment of left lower lobe, consistent with the CT finding. A decision was thus made to embolize the segmental bronchi using 7 mm Endobronchial Watanabe Spigot (*EWS* ^*®*^ *, Novatech, La Ciotat, France*) as an interim measure to prevent airway flooding while awaiting definitive treatment. A nylon thread was sutured beforehand to the distal end of the EWS for ease of forcep retrieval in the event of inadvertent grip-loss during insertion (Fig. 1). The forcep was introduced in advance into the therapeuptic bronchoscope, and the EWS grasped with the forcep. The bronchoscope with the grasped EWS was inserted through the endotracheal tube as a single unit, and inserted firmly into the target bronchi under direct vision (Fig. 2). Two 7 mm EWS were successfully embolized, one each into the lingula and postero-basal segment of left lower lobe. Total procedure time was 50 min. Post procedure CXR showed two EWS in optimal positions (Fig. 3). Subsequent bronchial artery embolization (BAE) attempt was abandoned after failure to identify the culprit vessels. In view of high surgical risk, patient was managed conservatively in the medical high dependency unit.

Patient was mechanically ventilated in ward with no recurrence of hemoptysis. She was transfused with one pint of packed cell, and haemoglobin level thereafter remained stable at 10.4 g/dl. A decision for EWS removal was made at day 4 of ventilation, as there was no evidence of re-bleeding; whereas there were concerns regarding her higher risk of infection due to underlying bronchiectasis, as well as possible deleterious effect on ventilation due to the lingular lobe EWS blockade in this elderly patient. Upon removal of EWS via flexible bronchoscope, the mucosa of the target bronchi was noted to be healthy with no purulent discharges or bleeding from the culprit bronchi. Despite the brief episode of cardiopulmonary resuscitation, she attained good neurological recovery and was extubated after EWS removal. She was discharged 2 days later with good functional status. Patient walked into our outpatient clinic 1 month later with no recurrence of hemoptysis and further investigation and management has since moved to focus on her underlying bronchiectasis. A summary and timeline of the clinical progress is as below (Table 1).

**Table 1:** Summary and timeline of clinical progress

Relevant past medical history and interventions			
78-year-old lady with no prior known chronic medical illness Non-smoker and no previous hospital admission			
Day	Summaries from initial and follow-up visits	Essential diagnostic testing	Interventions
Day 1	Massive hemoptysis and cardiac arrest	Haemoglobin level 13.5 g/dl Chest X-ray Bibasal alveolar infiltrates Thoracic CT angiogram Consolidation at posterior basal segment of left lower lobe and lingular Endotracheal tube aspirate Acid fast bacili—negative Xpert MTB/RIF—negative	Invasive mechanical ventilation Flexible bronchoscopy and EWS insertion Empirical broad spectrum antibiotic
	Post EWS insertion	Chest X-ray EWS in optimal position	Bronchial artery embolization—failed
Day 2	No recurrence of bleeding		
Day 3	No recurrence of bleeding	Haemoglobin level 7.5 g/dl	Transfuse 1 pint packed cell
Day 4	No recurrence of bleeding	Haemoglobin level 10.4 g/dl	Removal of EWS Extubated
Day 6	Ambulating in ward		Discharged from ward
Day 30	Follow up visit	Chest X-ray Bronchiectasis	Bronchiectasis management

## Discussion

The mortality rate from untreated massive hemoptysis is more than 50%, with cause of death usually due to asphyxia secondary to flooding of airways rather than exsanguination [4]. Hence, endobronchial embolization as an interim measure to prevent airway flooding is an essential step in the initial management of massive hemoptysis. Endobronchial embolization using EWS had shown promising results in the management of bronchopleural fistula with a technical success rate of 96.7% and a low complication rate [5]. This novel technique had since then been employed in the management of massive hemoptysis with a reported success rate of 78% in one series [3].

One of the main challenges in our case was the placement of EWS in the lingular bronchus. Ideally, EWS is designed for placement at the segmental or sub-segmental level to prevent significant ventilatory defect [5]; however, it is known that some segmental bronchi can be too small to allow the insertion of spigots [3]. Fortunately, post insertion CXR in our patient did not show any evidence of lobar collapse and there were no issues with mechanical ventilation. The second technical challenge was in the difficult anatomy of the bronchial tree—in this case, the anterior angulation of lingular bronchus. Various techniques had been reported to assist in this situation, which include the usage of guidewire and also the bidirectional guiding device used in radial endobronchial ultrasound [6, 7].

The EWS is usually employed as a temporary measure in the management of massive hemoptysis while awaiting surgery or bronchial artery embolization (BAE)—and our third challenge was the lack of conventional definitive therapy. An urgent BAE was arranged for our patient but was unfortunately not successful. In view of her elderly age with poor reserve and haemodynamic instability, she was deemed very high surgical risk. However, using EWS endobronchial embolization as a radical definitive treatment for hemoptysis had been reported in literature, and was proven successful in our case as well [8]. This is important as BAE service is not readily available in many regions and all massive hemoptysis patients are usually of high surgical risk in view of hemodynamic instability and multiple comorbidities.

The last clinical challenge we faced was deciding the duration of EWS placement for hemoptysis. Literature on the duration of EWS placement remains sparse and was mainly extrapolated from fistulous lung disease. There are no firm recommendations, as some reports did not observe any severe infections in patients with permanent placement [9]; whereas some suggest that temporary placement would be preferable in patients with risk factors predisposing to infection [10]. Decision for removal was made after 4 days in our patient, as we were concerned with her bronchiectatic risk of infection.

## Conclusions

In conclusion, endobronchial embolization with EWS—a procedure elegant in its innate simplicity—requiring only a flexible bronchoscope, a biopsy forceps, and of course the EWS itself—can play a great role in the prevention of airway flooding in massive hemoptysis. It is useful as a temporary measure before definitive therapy, or can itself be the main therapeutic player in the hemoptysis armament for patients at overly-high surgical risk, or when bronchial artery embolization services are not readily available. Our centre anticipates further experiences with this technique in the future.

## Abbreviations

EWS: Endobronchial Watanabe Spigot
CXR: chest X-ray
CT: computed tomography
BAE: bronchial artery embolization
